# Impact Responses and Wave Dissipation Investigation of a Composite Sandwich Shell Reinforced by Multilayer Negative Poisson’s Ratio Viscoelastic Polymer Material Honeycomb

**DOI:** 10.3390/ma17010233

**Published:** 2023-12-31

**Authors:** Xiaoqiang Zhou, Wanbiao Fu, Yun Wang, Hai Yan, Yicang Huang

**Affiliations:** 1 Department of Mechanics, School of Aerospace Engineering, Huazhong University of Science and Technology, Wuhan 430074, China; zhou_xq@hust.edu.cn (X.Z.); fuwb_george@163.com (W.F.); 2 Hubei Key Laboratory of Engineering Structural Analysis and Safety Assessment, Wuhan 430074, China; 3 Industrial Engineering and Research Institute, China Academy of Art, Hangzhou 310002, China20201089@caa.edu.cn (H.Y.); 4 R&D Center, Wuhan Second Ship Design and Research Institute, Wuhan 430205, China

**Keywords:** low-velocity impact, wave dissipation, viscoelastic polymer material, composite sandwich shells, negative Poisson’s ratio, hygrothermal effects

## Abstract

This analysis investigated the impact wave response and propagation on a composite sandwich shell when subjected to a low-velocity external shock, considering hygrothermal effects. The sandwich shell was crafted using face layers composed of functional gradient metal–ceramic matrix material and a core layer reinforced with negative Poisson’s honeycomb. The honeycomb layer consisted of a combination of viscoelastic polymer material and elastic material. The equivalent parameters for the functional gradient material in the face layers were determined using the Mori–Tanaka and Voigt models, and the parameters for the negative Poisson’s ratio honeycomb reinforcement core layer were obtained through Gibson’s unit cell model. Parameters relevant to a low-velocity impact were derived using a modified Hertz contact law. The internal deformations, strains, and stress of the composite sandwich shell were described based on the higher-order shear deformation theory. The dynamic equilibrium equations were established using Hamilton’s principle, and the Galerkin method along with the Newmark direct integration scheme was employed to calculate the shell’s response to impact. The validity of the analysis was confirmed through a comparison with published literature. This investigation showed that a multilayer negative Poisson’s ratio viscoelastic polymer material honeycomb-cored structure can dissipate impact wave energy swiftly and suppress shock effectively.

## 1. Introduction

Laminate structures find extensive applications in various engineering fields, including aerospace, aviation, military equipment, transportation, and mechanical engineering. This is primarily due to their exceptional characteristics, such as high specific strength and stiffness, low density, and remarkable design adaptability. As a result, the investigation of their mechanical behavior and structural design has garnered increasing interest among researchers and scholars [1,2,3,4,5]. Galos [1] presented a review on fiber reinforced polymer matrix composites formed using thin-ply laminates and Garg and Chalak [2] performed a critical review of the available literature for the prediction of the behavior of laminated composites and sandwich structures under hygrothermal conditions.

In the fields of materials science and solid mechanics, Poisson’s ratio serves as a fundamental indicator of the Poisson effect, which refers to the deformation experienced by a material in directions perpendicular to the specific direction of loading. It quantifies the relationship between transverse strain and axial strain. More precisely, Poisson’s ratio is defined as the negative ratio of transverse strain to axial strain. In simpler terms, it measures the extent of transversal elongation relative to axial compression when subjected to small changes. For isotropic materials, Poisson’s ratio has been proven to fall within the range of −1 to 0.5, based on thermodynamic considerations of strain energy. This theoretical framework provides a comprehensive understanding of the mechanical behavior exhibited by such materials [6]. However, for most solid materials, Poisson’s ratio typically falls within the range of 0 to 0.5. However, it is worth noting that in the case of soft materials like rubber, where the bulk modulus significantly surpasses the shear modulus, Poisson’s ratio tends to approach a value near 0.5 [7]. In the case of open-cell viscoelastic polymer material foams, Poisson’s ratio tends to approach zero due to the collapse of the foam cells under compression. This unique behavior can be attributed to the specific structure and mechanical properties of these foams. 

It is worth noting that many common solids found in nature typically exhibit Poisson’s ratios within the range of 0.2 to 0.3. This range is observed in a wide variety of traditional mechanical materials, highlighting their characteristic response to external forces. However, it is also important to acknowledge that certain artificial materials and microstructures have the potential to provide effective equivalent Poisson’s ratios that span the range between −1 and 1. These exceptional cases show the remarkable versatility and engineered capabilities of these materials in tailoring their mechanical properties to meet specific requirements [8]. These materials offer remarkable mechanical properties that are advantageous in various engineering applications. They demonstrate exceptional energy absorption capabilities and resistance to fractures, shear deformation, and indentation. To circumvent the need for a cumbersome expression, Evans et al. [9] proposed that materials with a negative Poisson’s ratio be referred to as “auxetic materials” or simply “auxetics.”

Negative Poisson’s ratio materials can be broadly classified into four categories: natural negative Poisson’s ratio materials, cellular negative Poisson’s ratio materials, metallic negative Poisson’s ratio materials, and multi-material negative Poisson’s ratio composites. The initial reports of materials with a negative Poisson’s ratio date back to the early 20th century, primarily observed in the realms of chemistry and biology. Notably, certain body-centered and face-centered cubic crystal minerals, as well as animal tissues, exhibited these intriguing characteristics [10]. In a study conducted by Lakes, the implications of negative Poisson’s ratios in component design under stress were investigated [11]. Their findings revealed that when the Poisson’s ratio assumes a negative value, stress concentration factors are reduced under certain conditions. This discovery sheds light on the potential benefits of incorporating negative Poisson’s ratio materials in the design process, as they can aid in minimizing stress concentration effects. Baughman et al. have astutely highlighted that negative Poisson’s ratios are a prevalent characteristic among cubic metals when oriented in specific directions [12]. Their research suggested that approximately 69% of cubic elemental metals exhibit a negative Poisson’s ratio when subjected to stretching along the (110) direction. Moreover, they have proposed several initial applications for such materials, including the use of negative Poisson’s ratio metals as electrodes in piezoelectric strain sensors and as vanes in aircraft gas turbine engines. These innovative applications demonstrate the potential utility of negative Poisson’s ratio materials in various technological domains.

Cellular negative Poisson’s ratio materials are classified as a type of porous material, which endows them with low density, exceptional mechanical energy absorption, noise reduction capabilities, and robust damping properties. In practical applications, common two-dimensional concave structures, including concave hexagonal honeycomb models, concave triangle models (also known as the double arrow model), and star models, have a negative Poisson’s ratio. It is important to note that the Poisson’s ratio characteristic of these concave structures is intricately linked to their unique concave angles. However, it is essential to recognize that the presence of internal concave–convex shapes alone is not enough to guarantee a negative Poisson’s ratio in the structure. Additional factors must be considered to ascertain the presence of a negative Poisson’s ratio. In the case of a star-shaped two-dimensional system, it is worth noting that auxetic angles below approximately 20 degrees do not result in a negative Poisson’s ratio. Moreover, an increase in the auxetic angle will lead to a reduction in the effective pure shear modulus, as highlighted in a comprehensive study [8]. To address these considerations, an analysis incorporating a concave hexagonal honeycomb structure can be employed to induce a negative Poisson’s ratio. In addition, to enhance mechanical energy dissipation and absorption, the introduction of viscoelastic damping materials has proven instrumental in this endeavor. Through this combined approach, the desired mechanical properties are amplified, offering potential benefits in a range of applications.

Functionally graded materials and/or structures exhibit a gradual variation in composition and structure throughout their volume, leading to consequential alterations in their properties. This unique characteristic allows for the intentional design and fabrication of materials and structures tailored to specific functions and applications. The concept of functionally graded materials and structures was initially introduced in the field of aerospace engineering, where the focus was on developing thermal barriers capable of withstanding exceedingly high temperatures. By strategically manipulating their composition and structure, these materials and structures can effectively serve as thermal barriers, shielding against extreme thermal conditions [13]. In engineering applications, the distribution of material gradients can be classified as either continuous or discontinuous. Functional gradient materials, exhibiting superior mechanical behaviors, are abundantly found in nature. For instance, plants showcase a distribution of fibers in bamboo and fruit peels, while animals exhibit a micro-porous distribution in bones and certain tissues. These natural occurrences serve as inspiration, highlighting the extensive presence of functional gradient materials and their potential for enhancing mechanical performance in practical engineering applications.

In the realm of practical engineering, the pursuit of specific mechanical behaviors has prompted the proposal and design of bioinspired functional gradient materials and microstructures. These innovative approaches aim to create system that can effectively absorb external loads, dissipate vibration energy within the structures, and resist external impacts. To fulfill these requirements, Zhou and Wang proposed a functional gradient structure with high stiffness in the outer layers and flexible inner layers. This ingenious design enables the structure to achieve its intended purpose of load absorption, energy dissipation, and impact resistance [14]. Furthermore, extensive investigations have been conducted on fiber-reinforced resin matrix materials with varying distribution configurations of functional gradient models. The insightful analysis conducted in these studies has unequivocally demonstrated that the implementation of a specified design for the functional gradient distribution of fibers can yield a remarkable increase in the buckling load capacity of structures. This finding underscores the significant impact that a well-designed functional gradient distribution can have on enhancing the structural integrity and load-bearing capabilities of the material. The potential for enhancing the buckling load through tailored functional gradient fiber distribution holds great promise in the field of engineering [14]. 

In the past few years, graphene platelets have emerged as a novel reinforcement material for enhancing the mechanical behavior of structures. Particularly, the concept of functionally graded designs in graphene-reinforced composite structures has garnered increasing interest from researchers and scientists. In this regard, Zhao et al. [15] have presented a comprehensive and critical review, shedding light on the advancements and potential of functionally graded graphene-reinforced composite structures. This review serves as a valuable resource for researchers and scientists who seek to delve deeper into this exciting area of study. Meanwhile, a monograph covering functional gradients inspired by biological material in terms of design principles, functions, and applications has been provided by Liu et al. [16]. FGM structures are extensively applied in mechanical engineering, such as in composite beams [17,18], plates [14,19], and shells [20,21]. 

As we know, there is a conflict between a material’s strength and toughness [22]. Ceramic matrix composite material with a functional gradient design is regarded as an efficient method to modify composites, which can achieve a material with extra high strength and toughness under high temperature, and have excellent microwave transparent and broadband wave transmission ability at the same time [23,24]. Sofiye [25] presented an exhaustive review of the literature on the vibration and buckling of functionally graded materials, especially for mechanical behavior investigations related to functionally graded and laminated sandwich conical shells. By applying the higher-order shear deformation theory and smeared stiffeners technique, assuming Coriolis acceleration and centrifugal forces, Aris and Ahmadi [20] derived a governing equation of a cross-stiffened, rotating, truncated conical shell with functional gradient materials and investigated the influences of the cone angle, volume fraction, and thermal conditions on the critical frequency. Assuming the thermo-mechanical characteristic was distributed by the temperature and geometric position of the structure, Bagheri et al. [26,27] constructed a functionally graded material assembled as a spherical conical shell and presented a series analysis under rapid heating through the generalized differential quadrature method and the Crank–Nicolson method. Except for thermal impedance, functionally graded materials show excellent behavior of sound transmission loss by a proper design. By applying the space harmonic approach and virtual work principle, Fu et al. [28] established a corrugated core functionally graded material sandwich plate filled with porous material and analytically investigated the sound transmission loss; meanwhile, their analysis was validated through published studies. Truncated conical shells are fundamental structures that are applied in engineering to increase the mechanical performance and functional grade, and are used to design stiffeners with different configurations. Considering complex loads, such as axial compressive loads and external uniform pressure, Dung and Chan [29] provided an analytical investigation of the buckling behaviors of truncated conical shells reinforced with orthogonal stiffeners. 

As recalled in the above literature, viscoelastic polymer materials are widely utilized in engineering for reducing and minimizing the sound radiation and vibration level by dissipation and absorbing mechanical energy propagation in composite structures. As all of us know, service conditions are the crucial effects that influence the parameters of viscoelastic polymer materials, such as Young’s modulus, damping ratio, and Poison’s ratio. To accurately evaluate the dynamic behavior of composite structures, a number of researchers have investigated the thermal and moisture behaviors of viscoelastic polymer materials [30,31]. In order to eliminate power machinery vibration and noise, Qu et al. [32] prepared a novel semi-interpenetrating viscoelastic material through linear polyimide and crosslinked epoxy, and investigated the damping, thermal, and mechanical performances of this new viscoelastic composite. By using the representative volume element mode with a random distribution of carbon fibers, Yuan et al. [33] proposed a temperature-dependent micromechanical model to predict the temperature-dependent mechanical behaviors of this composite, such as the transverse tensile strength and transverse compressive strength, failure behaviors, and parametric sensitivity. Moreover, the moisture and humidity of the environment influence the mechanical parameters of viscoelastic composites. Under a complex external load, the mechanisms of moisture intrusion, water penetration, and diffusion in viscoelastic composites has attracted a lot of investigation [34,35]. Ye and Zhang [35] established a thermodynamically consistent moisture diffusion model for coupling the moisture diffusion effect and viscoelastic response of the multiphase viscoelastic composites, developed a two-constituent phase-field fracture model for describing the hygroscopic swelling behaviors at the multiphase interfacial decohesion of viscoelastic polymer material, and proposed a crack filter theory for characterizing the moisture flux propagation at the interface between the multiphases. In fact, the hardening and softening behaviors of viscoelastic polymer materials are influenced by other factors, such as the strain rate, frequency, duration, and external load amplitude [36,37,38,39,40]. Shitikova and Krusser [39] presented a comprehensive review on the historical development and formulation of viscoelastic materials’ models in application and investigation, which mainly covers the classical models and fractional model. Barba et al. [36] provided experimental and modeling insights into the mechanical properties of PEEK under different strain rates and temperatures.

To achieve a balance between high strength and toughness while enhancing temperature resistance, a functional gradient design method was employed for a ceramic matrix with a metal-filled composite. Simultaneously, a composite reinforced layer with a negative Poisson’s ratio was created using a viscoelastic polymer material and elastic honeycomb reinforcement. Investigations in the present work are outlined as follows: the problem is provided, and then mathematical modeling, derivation processing, equivalent performance, and governing equation construction are proposed in Section 2; a determination of the response and wave propagation behaviors of the composite laminated shells is presented in detail in Section 3; numerical results and a qualitative discussion are provided in Section 4, and a conclusion is given in Section 5.

## 2. Mathematical Modeling

**Figure 1:**
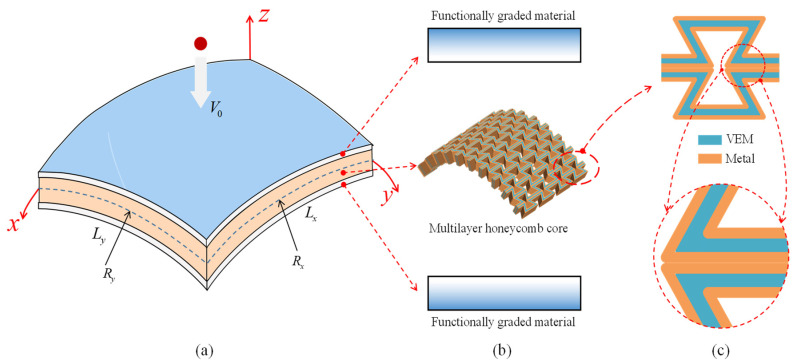
Schematic diagram of the composite sandwich shell and its constituents: (**a**) the structure under a low-velocity impact, (**b**) three layers of the structure; and (**c**) constituents of the multilayer honeycomb core.

In this paper, we present the design of a novel functionally graded composite sandwich doubly curved shell. The core of this structure features a multilayer honeycomb with a negative Poisson’s ratio, as illustrated in Figure 1. The face-sheets of the shell are composed of functionally graded materials, combining ceramic and metal layers. The honeycomb core consists of three layers, with the middle layer being a viscoelastic polymer material and the inner and outer layers being solid metal. To investigate the behavior of the composite sandwich shell, it is subjected to impact by a sphere with a low velocity *V*_0_ in hygrothermal environments. The structure is defined by a global coordinate system (*x*, *y*, *z*) with the mid-plane set at *z* = 0. The principal curvature radiuses at the mid-plane are denoted as *R_x_* and *R_y_* along the *x* and *y* axes, respectively. The lengths of the curved edge in the *x* and *y* directions are represented by *L_x_* and *L_y_*, respectively. Both the top and bottom face-sheets exhibit symmetry about the mid-plane, with an equivalent thickness denoted as *h_f_*_._ The core layer of the structure possesses a thickness of *h_c_*. Each layer is bonded together well, and there is no sliding during the impact.

Considering the remarkable properties exhibited by ceramics, such as high strength, exceptional hardness, and excellent temperature resistance, the face-sheets of the structure are fabricated using functionally graded materials that transition from ceramic to metal from the exterior to the interior. The composition of the face-sheets is assumed to vary continuously throughout the thickness of the structure, following a power law distribution. The volume fraction of ceramic within the face-sheets can be mathematically expressed as:

(1) $$V_{c}\left(z\right)=\left\{\begin{array}{cc} {\left(\frac{z-h_{c}/2}{h_{f}}\right)}^{p} & h_{c}/2\leq z\leq h/2\\ {\left(\frac{-z-h_{c}/2}{h_{f}}\right)}^{p} & -h/2\leq z\leq -h_{c}/2 \end{array}\right.$$

The volume fraction of metal can be deduced using a complementary approach as:

(2) $$V_{m}\left(z\right)=1-V_{c}\left(z\right)$$

**Figure 2:**
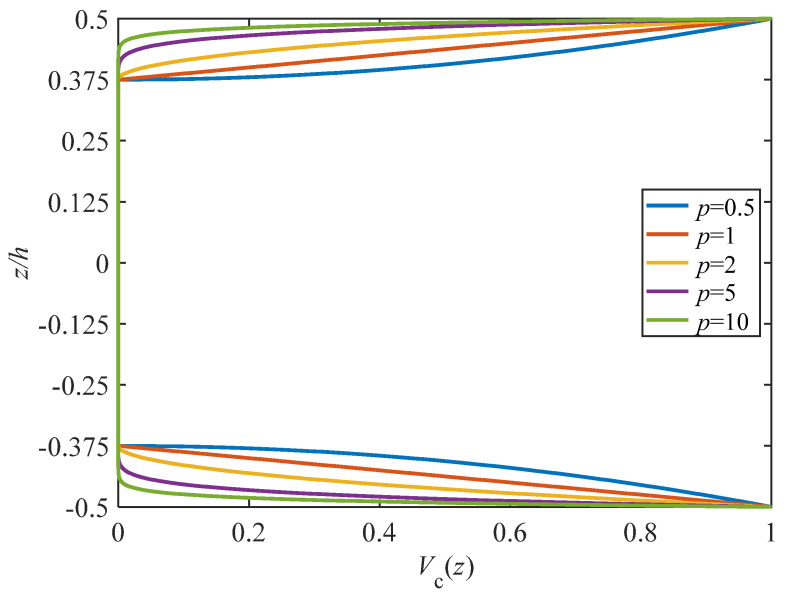
The volume fraction of ceramic in face-sheets for different values of the power law index *p*.

The power law index, denoted as *p*, and the total thickness of the structure, represented by *h* (where *h* = 2*h_f_* + *h_c_*), play crucial roles in determining the volume fraction of ceramic along the *z* direction. Figure 2 presents the volume fraction of ceramic for various values of the power law index *p*. As one moves from the inner surface to the outer surface of the face-sheets, the volume fraction of ceramic, *V_c_*, increases continuously from 0 to 1. Notably, it is evident that a decrease in the value of *p* leads to an increase in the volume fraction of ceramic.

### 2.1. Equivalent Mechanical Parameters

Before proceeding with further derivation, it is necessary to determine the equivalent mechanical properties of the sandwich shell. The face-sheets, being composed of functionally graded materials, can have their equivalent mechanical properties obtained using the law of mixture. This enables us to calculate the overall properties of the face-sheets by considering the distribution and composition of the constituent materials.

(3) $$P\left(z\right)=\left(P_{c}-P_{m}\right)V_{c}\left(z\right)+P_{m}$$

where *P*(*z*) represents the equivalent mechanical properties, such as Young’s modulus, shear modulus, mass density, Poisson’s ratio, and more; and the subscripts c and m denote the ceramic and metal constituents, respectively. Considering the influence of temperature variation on the mechanical properties of both ceramic and metal, the temperature-dependence of the material properties can be expressed using the following relationships [41]:

(4) $$P=P_{0}\left(1+P_{1}T+P_{2}T^{2}+P_{3}T^{3}\right)$$

where *P*_0_, *P*_1_, *P*_2_, and *P*_3_ are the temperature-dependent coefficients. For materials Si_3_N_4_ and SUS304, the temperature-dependent coefficients are listed in Table 1.

**Figure 3:**
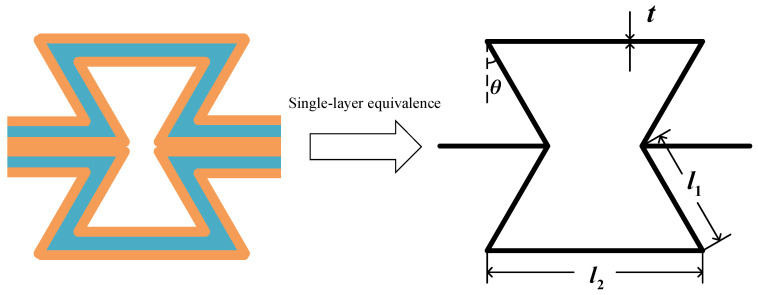
The single-layer equivalence for the multilayer negative Poisson’s ratio honeycomb with viscoelastic polymer material.

To obtain the equivalent modulus of the core layer, the multilayer honeycomb walls should be firstly replaced with a single equivalent layer, as shown in Figure 3, and then the analytical expressions derived by Gibson et al. [43] can be used. Based on the classical lamination theory, the relationship between the forces and strains of multilayer structures can be expressed as:

(5) $$\left\{\begin{array}{c} N\\ M \end{array}\right\}=\left[\begin{array}{cc} A & B\\ B^{T} & D \end{array}\right]\left\{\begin{array}{c} \varepsilon^{0}\\ \kappa \end{array}\right\}$$

The extensional stiffness, coupling stiffness, and bending stiffness matrices, denoted as *A*, *B*, and *D*, respectively, play a crucial role in characterizing the mechanical behavior of the structure. The expressions for these matrices are as follows:

(6) $$\left[A_{ij},B_{ij},D_{ij}\right]=\sum_{n=1}^{3}\int_{z_{n-1}}^{z_{n}}Q_{ij}^{\left(n\right)}\left[1,z,z^{2}\right]dz\;i,j=1,2,6$$

Equation (5) can be reformulated as follows:

(7) $$\left\{\begin{array}{c} \varepsilon^{0}\\ \kappa \end{array}\right\}=\left[\begin{array}{cc} a & b\\ b^{T} & d \end{array}\right]\left\{\begin{array}{c} N\\ M \end{array}\right\}$$

In accordance with the concept of Young’s modulus, let us assume that the structure experiences solely normal strain with no shear strains, denoted as *κ* = 0. Under this assumption, the expression simplifies to the following:

(8) $$M=-d^{-1}b^{T}N$$

(9) $$\varepsilon^{0}=aN+bM=\left(a-bd^{-1}b^{T}\right)N=PN$$

Writing Equation (9) in its explicit form as follows:

(10) $$\left\{\begin{array}{c} \varepsilon_{xx}^{0}\\ \varepsilon_{yy}^{0}\\ \gamma_{xy}^{0} \end{array}\right\}=\left[\begin{array}{ccc} P_{11} & P_{12} & P_{16}\\ & P_{22} & P_{26}\\ sym & & P_{66} \end{array}\right]\left\{\begin{array}{c} N_{xx}\\ N_{yy}\\ N_{xy} \end{array}\right\}$$

Due to the symmetry of matrices *A*, *B,* and *D*, the resulting matrix *P* is also symmetric. To obtain the equivalent axial modulus, the multilayer structure must be placed in a unidirectional tensile state. To achieve this, an additional shear force is applied to counteract any shear deformation. To ensure $\gamma_{\mathrm{xy}}^{0}$ = 0, the needed shear force:

(11) $$N_{xy}=-\left(P_{16}N_{xx}+P_{26}N_{yy}\right)/P_{66}$$

Substituting Equation (11) into Equation (10), according to the stress–strain relationship, the in-plane axial moduli can be expressed as:

(12) $$E_{xx}=\frac{1}{\left(P_{11}-P_{16}^{2}/P_{66}\right)t}$$

(13) $$E_{yy}=\frac{1}{\left(P_{22}-P_{26}^{2}/P_{66}\right)t}$$

where *t* represents the total thickness of the multilayer structure. The equivalent shear modulus can be expressed as follows when the structure is subjected to a pure shear state:

(14) $$G_{xy}=\frac{1}{\left(P_{66}-\frac{1}{\Delta_{1}}\left(P_{16}^{2}P_{22}-2P_{26}P_{16}+P_{26}^{2}P_{11}\right)\right)t},\;\Delta_{1}=P_{11}P_{22}-P_{12}^{2}$$

The equivalent Poisson’s ratio can be determined as:

(15) $$\nu_{xy}=-\frac{P_{12}-P_{13}P_{26}/P_{66}}{P_{11}-P_{16}^{2}/P_{66}}$$

Then we can calculate the equivalent mechanical properties of the multilayer honeycomb core based on the expressions [44]:

(16) $$E_{11}=E\frac{\eta_{3}^{3}\left(\eta_{1}-\sin\theta \right)}{\cos\theta \left(1+\left(\tan^{2}\theta +\eta_{1}\sec^{2}\theta \right)\eta_{3}^{2}\right)}$$

(17) $$E_{22}=E\frac{\eta_{3}^{3}}{\cos\theta \left(\eta_{1}-\sin\theta \right)\left(\tan^{2}\theta +\eta_{3}^{2}\right)}$$

(18) $$G_{12}=G\frac{\eta_{3}^{3}}{\eta_{1}\left(1+2\eta_{1}\right)\cos\theta }$$

(19) $$G_{23}=G\frac{\eta_{3}\cos\theta }{\eta_{1}-\sin\theta }$$

(20) $$G_{13}=G\frac{\eta_{3}}{2\cos\theta }\left(\frac{\eta_{1}-\sin\theta }{1+2\eta_{1}}+\frac{\eta_{1}+2\sin^{2}\theta }{2\left(\eta_{1}-\sin\theta \right)}\right)$$

(21) $$\nu_{12}=-\frac{\sin\theta \left(1-\eta_{3}^{2}\right)\left(\eta_{1}-\sin\theta \right)}{\cos^{2}\theta \left(1+\left(\tan^{2}\theta +\eta_{1}\sec^{2}\theta \right)\eta_{3}^{2}\right)}$$

(22) $$\nu_{21}=-\frac{\sin\theta \left(1-\eta_{3}^{2}\right)}{\left(\tan^{2}\theta +\eta_{3}^{2}\right)\left(\eta_{1}-\sin\theta \right)}$$

(23) $$\rho =\rho \frac{\eta_{3}\left(\eta_{1}+2\right)}{2\cos\theta \left(\eta_{1}-\sin\theta \right)}$$

(24) $$\alpha_{11}=\alpha \frac{\eta_{3}\cos\theta }{\sin\theta +\alpha }$$

(25) $$\alpha_{22}=\alpha \frac{\eta_{3}\left(\eta_{1}+\sin\theta \right)}{\left(2\eta_{1}+1\right)\cos\theta }$$

where *θ* is the inclined angle, and *η*_1_ and *η*_3_ are two dimensionless parameters related to the geometry of the honeycomb, *η*_1_ = *l*_2_/*l*_1_, *η*_3_ = *t*/*l*_1_.

### 2.2. Impact Forces Determination

For analyzing the dynamic responses of this composite laminated shell, it is imperative to determine the transient impact load acting on the composite laminate shells. Considering the indentation period and contact area between the impacting body and the composite laminate, this contacting load can be regarded as a force varying over time. This force can be expressed as:

(26) $$F=F_{c}\delta \left(x-x_{c}\right)\delta \left(y-y_{c}\right)$$

where *F*_c_ denotes the impact force between the indenter and the composite laminate shell, *δ* denotes the Dirac delta function, and (*x_c_*, *y_c_*) defines the coordinates of the indentation center. For our investigation, we have selected the central point of the sandwich, denoted by (*L_x_*/2, *L_y_*/2). The impact force is determined through the modified Hertz contact law. In this analysis, two stages are defined, and the contact force can be obtained by applying the following equation [45]:

(27) $$F_{c}=\left\{\begin{array}{cc} k_{c}\alpha^{1.5} & \mathrm{Loading}\;\mathrm{stage}\\ F_{c}^{\max}{\left(\frac{\alpha -\alpha_{0}}{\alpha_{\max}-\alpha_{0}}\right)}^{2.5} & \;\mathrm{Unloading}\;\mathrm{stage} \end{array}\right.$$

The modified Hertz contact stiffness, denoted as *k_c_*, plays a crucial role in this analysis. By utilizing this stiffness, we can determine the indentation *α* as follows:

(28) $$\alpha =\bar{w}\left(t\right)-w_{0}\left(x_{c},y_{c},t\right)$$

**Figure 4:**
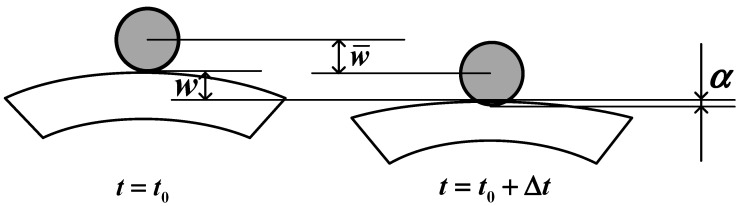
A schematic of the indentation process.

In the above equation, $\bar{w}$(*t*) expresses the displacement at the contact point in the transient analysis, and *w*_0_ expresses the deflection of the composite sandwich shell just at the point where the impact contacts the impactor, as shown in Figure 4.

In Equation (27), *α*_max_ and $F_{c}^{\max}$ express the maximum indentation and contacting force in the loading procedure, and *α*_0_ denotes the permanent indentation generated by the impact load. In the present analysis, permanent damage to the composite sandwich shell under a low-velocity impact is not taken into consideration, and thus it is assumed that *α*_0_ is equal to zero. According to Ref. [46], the modified Hertz contact stiffness *k*_c_ can be yielded according the following equation:

(29) $$k_{c}=\frac{4}{3}E^{*}\sqrt{R_{\mathrm{imp}}^{}}$$

(30) $$E^{*}=\frac{E_{\mathrm{imp}}E_{22}}{E_{\mathrm{imp}}\left(1-\nu_{22}^{2}\right)+E_{22}\left(1-\nu_{\mathrm{imp}}^{2}\right)}$$

where *E*_imp_ is Young’ modulus, *ν*_imp_ denotes Poisson’s ratio, *R*_imp_ means the impactor’s radius, and *ν_2_*_2_ and *E*_22_ express the Poisson’s ratio and Young’ modulus of the top and bottom layers of the functional gradient sandwich structure, respectively.

### 2.3. Constitutive Equations and In-Plane Variables Determination

According to the higher-order shear deformation theory [47], the displacement of the functional gradient sandwich shell can be defined as:

(31) $$\begin{array}{l} u\left(x,y,z,t\right)=\left(1+\frac{z}{R_{x}}\right)u_{0}\left(x,y,t\right)-z\frac{\partial w_{0}\left(x,y,t\right)}{\partial x}+f\left(z\right)\phi_{x}\left(x,y,t\right)\\ v\left(x,y,z,t\right)=\left(1+\frac{z}{R_{y}}\right)v_{0}\left(x,y,t\right)-z\frac{\partial w_{0}\left(x,y,t\right)}{\partial y}+f\left(z\right)\phi_{y}\left(x,y,t\right)\\ w\left(x,y,z,t\right)=w_{0}\left(x,y,t\right) \end{array}$$

where *u*_0_, *v*_0,_ and *w*_0_ are the displacement variables at the mid-plane of the laminated sandwich shell; *ϕ_x_* and *ϕ_y_* express the rotations of the normal to mid-plane about the *y* and *x* axes; and function *f*(*z*) determines the distribution of the transverse shear strains along the shell thickness. A lot of different functions can be applied. In this work, the cubic function was selected as follows:

(32) $$f\left(z\right)=z-\frac{4}{3}\frac{z^{3}}{h^{2}}$$

where *h* is the total thickness of the functional gradient laminate structure, *h* = 2*h*_f_ + *h*_c_.

According to the strain displacement relationships of the shell structure:

(33) $$\begin{array}{l} \varepsilon_{xx}=\frac{\partial u}{\partial x}+\frac{w}{R_{x}},\;\varepsilon_{yy}=\frac{\partial v}{\partial y}+\frac{w}{R_{y}},\;\varepsilon_{zz}=\frac{\partial w}{\partial z}=0\\ \gamma_{xy}=\frac{\partial v}{\partial x}+\frac{\partial u}{\partial y},\;\gamma_{xz}=\frac{\partial w}{\partial x}+\frac{\partial u}{\partial z}-\frac{u_{0}}{R_{x}},\;\gamma_{yz}=\frac{\partial v}{\partial z}+\frac{\partial w}{\partial y}-\frac{v_{0}}{R_{y}} \end{array}$$

All nonzero strain components have the form:

(34) $$\left\{\begin{array}{c} \varepsilon_{xx}\\ \varepsilon_{yy}\\ \gamma_{xy} \end{array}\right\}=\left\{\begin{array}{c} \varepsilon_{xx}^{0}\\ \varepsilon_{yy}^{0}\\ \gamma_{xy}^{0} \end{array}\right\}+z\left\{\begin{array}{c} \varepsilon_{xx}^{1}\\ \varepsilon_{yy}^{1}\\ \gamma_{xy}^{1} \end{array}\right\}+f\left(z\right)\left\{\begin{array}{c} \varepsilon_{xx}^{s}\\ \varepsilon_{yy}^{s}\\ \gamma_{xy}^{s} \end{array}\right\},\;\left\{\begin{array}{c} \gamma_{yz}\\ \gamma_{xz} \end{array}\right\}=f^{\prime }\left(z\right)\left\{\begin{array}{c} \gamma_{yz}^{s}\\ \gamma_{xz}^{s} \end{array}\right\}$$

where the strains in-plane can be expressed as:

(35) $$\left\{\begin{array}{c} \varepsilon_{xx}^{0}\\ \varepsilon_{yy}^{0}\\ \gamma_{xy}^{0} \end{array}\right\}=\left\{\begin{array}{c} \frac{\partial u_{0}}{\partial x}+\frac{w_{0}}{R_{x}}\\ \frac{\partial v_{0}}{\partial y}+\frac{w_{0}}{R_{y}}\\ \frac{\partial v_{0}}{\partial x}+\frac{\partial u_{0}}{\partial y} \end{array}\right\},\;\left\{\begin{array}{c} \varepsilon_{xx}^{1}\\ \varepsilon_{yy}^{1}\\ \gamma_{xy}^{1} \end{array}\right\}=\left\{\begin{array}{c} \frac{1}{R_{x}}\frac{\partial u_{0}}{\partial x}-\frac{\partial^{2}w_{0}}{\partial x^{2}}\\ \frac{1}{R_{y}}\frac{\partial v_{0}}{\partial y}-\frac{\partial^{2}w_{0}}{\partial y^{2}}\\ \frac{1}{R_{x}}\frac{\partial u_{0}}{\partial y}+\frac{1}{R_{y}}\frac{\partial v_{0}}{\partial x}-2\frac{\partial^{2}w_{0}}{\partial x\partial y} \end{array}\right\},\;\left\{\begin{array}{c} \varepsilon_{xx}^{s}\\ \varepsilon_{yy}^{s}\\ \gamma_{xy}^{s} \end{array}\right\}=\left\{\begin{array}{c} \frac{\partial \phi_{x}}{\partial x}\\ \frac{\partial \phi_{y}}{\partial y}\\ \frac{\partial \phi_{y}}{\partial x}+\frac{\partial \phi_{x}}{\partial y} \end{array}\right\}$$

And meanwhile, the transverse shear strains can be denoted as:

(36) $$\left\{\begin{array}{c} \gamma_{yz}^{s}\\ \gamma_{xz}^{s} \end{array}\right\}=\left\{\begin{array}{c} \phi_{y}\\ \phi_{x} \end{array}\right\}$$

The constitutive relationship for the *n*th layer of these functional gradient composite sandwich shell structures can be expressed as:

(37) $${\left\{\sigma \right\}}^{\left(n\right)}={\left[Q\right]}^{\left(n\right)}{\left\{\varepsilon \right\}}^{\left(n\right)}$$

In the above equations, [**Q**]^(*n*)^ expresses the matrices of stiffness, {**σ**}^(*n*)^ is the stress, and {**ε**}^(*n*)^ denotes the strain vectors of the composite sandwich shell at the layer *n*th, respectively. The variables in Equation (37) can be expressed as:

(38) $${\left\{\begin{array}{c} \sigma_{xx}\\ \sigma_{yy}\\ \sigma_{yz}\\ \sigma_{xz}\\ \sigma_{xy} \end{array}\right\}}^{\left(n\right)}={\left[\begin{array}{ccccc} Q_{11} & Q_{12} & 0 & 0 & 0\\ Q_{21} & Q_{22} & 0 & 0 & 0\\ 0 & 0 & Q_{44} & 0 & 0\\ 0 & 0 & 0 & Q_{55} & 0\\ 0 & 0 & 0 & 0 & Q_{66} \end{array}\right]}^{\left(n\right)}{\left\{\begin{array}{c} \varepsilon_{xx}-\varepsilon_{xx}^{\mathrm{HT}}\\ \varepsilon_{yy}-\varepsilon_{yy}^{\mathrm{HT}}\\ \gamma_{yz}\\ \gamma_{xz}\\ \gamma_{xy} \end{array}\right\}}^{\left(n\right)}$$

The coefficients of the equivalent stiffness are dependent on the Young’s modulus and Poisson’s ratio of the materials. For the functionally graded material face-sheets, the coefficients *Q_ij_* are also related to the space coordinates, which are:

(39) $$Q_{11}^{\left(n\right)}=Q_{22}^{\left(n\right)}=\frac{E^{\left(n\right)}\left(z\right)}{1-{\left(\nu^{\left(n\right)}\left(z\right)\right)}^{2}},\;Q_{12}^{\left(n\right)}=Q_{21}^{\left(n\right)}=\frac{\nu^{\left(n\right)}\left(z\right)E^{\left(n\right)}\left(z\right)}{1-{\left(\nu^{\left(n\right)}\left(z\right)\right)}^{2}},\;Q_{44}^{\left(n\right)}=Q_{55}^{\left(n\right)}=Q_{66}^{\left(n\right)}=\frac{E^{\left(n\right)}\left(z\right)}{2\left(1+\nu^{\left(n\right)}\left(z\right)\right)}$$

For the negative Poisson’s ratio core layer of the composite structure, the coefficients *Q_ij_* are:

(40) $$Q_{11}^{\left(n\right)}=\frac{E_{11}^{\left(n\right)}}{1-\nu_{12}^{\left(n\right)}\nu_{21}^{\left(n\right)}},\;Q_{22}^{\left(n\right)}=\frac{E_{22}^{\left(n\right)}}{1-\nu_{12}^{\left(n\right)}\nu_{21}^{\left(n\right)}},\;Q_{12}^{\left(n\right)}=Q_{21}^{\left(n\right)}=\nu_{12}^{\left(n\right)}Q_{22}^{\left(n\right)},\;Q_{44}^{\left(n\right)}=G_{23}^{\left(n\right)},\;Q_{55}^{\left(n\right)}=G_{13}^{\left(n\right)},\;Q_{66}^{\left(n\right)}=G_{12}^{\left(n\right)}$$

In Equation (38), two parameters, $\varepsilon_{xx}^{\mathrm{HT}}$ and $\varepsilon_{yy}^{\mathrm{HT}}$ are strains defined to describe the hygrothermal effects, and those two variables can be determined according to the following equations:

(41) $$\varepsilon_{xx}^{\mathrm{HT}}=\alpha_{11}\left(T-T_{0}\right)+\beta_{11}\left(H-H_{0}\right)$$

(42) $$\varepsilon_{yy}^{\mathrm{HT}}=\alpha_{22}\left(T-T_{0}\right)+\beta_{22}\left(H-H_{0}\right)$$

In Equations (41) and (42), *α*_11_ and *α*_22_ are coefficients of thermal, and *β*_11_ and *β*_22_ are moisture expansions in 11 and 22 directions, respectively; *T* means temperature and *H* means moisture. In the present research, three different distribution patterns of temperature and moisture along the thickness of the structure are considered.

Uniform distribution (UD):

(43) $$T\left(z\right)=T_{0}+\Delta T,\;H\left(z\right)=H_{0}+\Delta H$$

Linear distribution (LD):

(44) $$T\left(z\right)=T_{0}+\Delta T\left(\frac{1}{2}+\frac{z}{h}\right),\;H\left(z\right)=H_{0}+\Delta H\left(\frac{1}{2}+\frac{z}{h}\right)$$

Sinusoidal distribution (SD):

(45) $$T\left(z\right)=T_{0}+\Delta T\left(1-\cos\left(\frac{\pi }{2}\left(\frac{1}{2}+\frac{z}{h}\right)\right)\right),\;H\left(z\right)=H_{0}+\Delta H\left(1-\cos\left(\frac{\pi }{2}\left(\frac{1}{2}+\frac{z}{h}\right)\right)\right)$$

In Equations (43)–(45), *T*_0_ denotes the reference temperature and *H*_0_ expresses moisture, respectively. In the presented analysis, *T*_0_ = 300 K and *H*_0_ = 0 wt% are assumed.

### 2.4. Dynamic Equilibrium Equations

For this functional gradient composite sandwich shell, according to Hamilton’s principle, the dynamic equilibrium equations can be achieved as:

(46) $$\int_{t_{0}}^{t_{1}}\delta Ldt=\int_{t_{0}}^{t_{1}}\left(\delta U+\delta V-\delta K\right)dt=0$$

where *δU* means virtual strain energy, *δV* the virtual work done by external forces, and *δK* describes the virtual kinetic energy.

The virtual kinetic energy of this functional gradient composite sandwich shell can be achieved as:

(47) $$\delta K=\sum_{n=1}^{3}\int_{V^{\left(n\right)}}\rho^{\left(n\right)}\left(\dot{u}\delta \dot{u}+\dot{v}\delta \dot{v}+\dot{w}\delta \dot{w}\right)dV$$

By substituting Equation (31) into Equation (47), we can determine the virtual kinetic energy as:

(48) $$\delta K=\int_{S}\rho^{\left(n\right)}\left\{\begin{array}{l} -J_{x0}{\ddot{u}}_{0}\delta u_{0}-J_{x1}\frac{\partial {\ddot{u}}_{0}}{\partial x}\delta w_{0}-J_{x3}{\ddot{u}}_{0}\delta \phi_{x}+J_{x1}\frac{\partial {\ddot{w}}_{0}}{\partial x}\delta u_{0}+I_{2}\frac{\partial^{2}{\ddot{w}}_{0}}{\partial x^{2}}\delta w_{0}\\ +I_{4}\frac{\partial {\ddot{w}}_{0}}{\partial x}\delta \phi_{x}-J_{x3}{\ddot{\phi }}_{x}\delta u_{0}-I_{4}\frac{\partial {\ddot{\phi }}_{x}}{\partial x}\delta w_{0}-I_{5}{\ddot{\phi }}_{x}\delta \phi_{x}-J_{y0}{\ddot{v}}_{0}\delta v_{0}\\ -J_{y1}\frac{\partial {\ddot{v}}_{0}}{\partial y}\delta w_{0}-J_{y3}{\ddot{v}}_{0}\delta \phi_{y}+J_{y1}\frac{\partial {\ddot{w}}_{0}}{\partial y}\delta v_{0}+I_{2}\frac{\partial^{2}{\ddot{w}}_{0}}{\partial y^{2}}\delta w_{0}\\ +I_{4}\frac{\partial {\ddot{w}}_{0}}{\partial y}\delta \phi_{y}-J_{y3}{\ddot{\phi }}_{y}\delta v_{0}-I_{4}\frac{\partial {\ddot{\phi }}_{y}}{\partial y}\delta w_{0}-I_{5}{\ddot{\phi }}_{y}\delta \phi_{y}-{\ddot{w}}_{0}\delta w_{0} \end{array}\right\}dS$$

where the mass inertias can be defined as:

(49) $$\left[I_{0},I_{1},I_{2},I_{3},I_{4},I_{5}\right]=\sum_{n=1}^{3}\int_{z_{n-1}}^{z_{n}}\rho^{\left(n\right)}\left[1,z,z^{2},f\left(z\right),zf\left(z\right),f^{2}\left(z\right)\right]dS$$

(50) $$J_{\alpha 0}=I_{0}+\frac{2}{R_{\alpha }}I_{1}+\frac{1}{R_{\alpha }^{2}}I_{2}\left(\alpha =x,y\right)$$

(51) $$J_{\alpha i}=I_{i}+\frac{1}{R_{\alpha }}I_{i+1}\left(\alpha =x,y;\;i=1,3\right)$$

Furthermore, the virtual strain energy of the functional gradient composite sandwich shell can be achieved as:

(52) $$\begin{array}{c} \delta U=\sum_{n=1}^{3}\int_{V^{\left(n\right)}}\left(\sigma_{xx}^{\left(n\right)}\delta \varepsilon_{xx}^{\left(n\right)}+\sigma_{yy}^{\left(n\right)}\delta \varepsilon_{yy}^{\left(n\right)}+\sigma_{xy}^{\left(n\right)}\delta \gamma_{xy}^{\left(n\right)}+\sigma_{xz}^{\left(n\right)}\delta \gamma_{xz}^{\left(n\right)}+\sigma_{yz}^{\left(n\right)}\delta \gamma_{yz}^{\left(n\right)}\right)dV\\ \;\;=\int_{S}\left\{\begin{array}{l} N_{xx}\delta \varepsilon_{xx}^{0}+M_{xx}\delta \varepsilon_{xx}^{1}+P_{xx}\delta \varepsilon_{xx}^{s}+N_{yy}\delta \varepsilon_{yy}^{0}+M_{yy}\delta \varepsilon_{yy}^{1}+P_{yy}\delta \varepsilon_{yy}^{s}\\ +N_{xy}\delta \gamma_{xy}^{0}+M_{xy}\delta \gamma_{xy}^{1}+P_{xy}\delta \gamma_{xy}^{s}+S_{yz}\delta \gamma_{yz}^{s}+S_{xz}\delta \gamma_{xz}^{s} \end{array}\right\}dS \end{array}$$

Furthermore, the forces and moments variables of the sandwich can be obtained as:

(53) $$\left[N_{i},M_{i},P_{i}\right]=\sum_{n=1}^{3}\int_{z_{n-1}}^{z_{n}}\sigma_{i}^{\left(n\right)}\left[1,z,f\left(z\right)\right]dz\;\left(i=xx,yy,xy\right)$$

(54) $$S_{j}=\sum_{n=1}^{3}\int_{z_{n-1}}^{z_{n}}\sigma_{j}^{\left(n\right)}f^{\prime }\left(z\right)dz\;\left(j=yz,xz\right)$$

Moreover, the above resultants can be also arranged as:

(55) $$\begin{array}{l} \left\{\begin{array}{l} N_{xx}\\ N_{yy}\\ N_{xy}\\ M_{xx}\\ M_{yy}\\ M_{xy}\\ P_{xx}\\ P_{yy}\\ P_{xy} \end{array}\right\}=\left[\begin{array}{ccccccccc} A_{11} & A_{12} & 0 & B_{11} & B_{12} & 0 & B_{11}^{s} & B_{12}^{s} & 0\\ & A_{22} & 0 & B_{12} & B_{22} & 0 & B_{12}^{s} & B_{22}^{s} & 0\\ & & A_{66} & 0 & 0 & B_{66} & 0 & 0 & B_{66}^{s}\\ & & & D_{11} & D_{12} & 0 & D_{11}^{s} & D_{12}^{s} & 0\\ & & & & D_{22} & 0 & D_{12}^{s} & D_{22}^{s} & 0\\ & & & & & D_{66} & 0 & 0 & D_{66}^{s}\\ & & & & & & H_{11}^{s} & H_{12}^{s} & 0\\ & & & & & & & H_{22}^{s} & 0\\ sym. & & & & & & & & H_{66}^{s} \end{array}\right]\left\{\begin{array}{l} \varepsilon_{xx}^{0}\\ \varepsilon_{yy}^{0}\\ \gamma_{xy}^{0}\\ \varepsilon_{xx}^{1}\\ \varepsilon_{yy}^{1}\\ \gamma_{xy}^{1}\\ \varepsilon_{xx}^{s}\\ \varepsilon_{yy}^{s}\\ \gamma_{xy}^{s} \end{array}\right\} \end{array}$$

(56) $$\left\{\begin{array}{c} S_{yz}\\ S_{xz} \end{array}\right\}=\left[\begin{array}{cc} A_{44}^{s} & 0\\ 0 & A_{55}^{s} \end{array}\right]\left\{\begin{array}{c} \gamma_{yz}^{s}\\ \gamma_{xz}^{s} \end{array}\right\}$$

The stiffness coefficients of the functional gradient composite sandwich shell can be determined by:

(57) $$\left[A_{ij},B_{ij},D_{ij},A_{ij}^{s},B_{ij}^{s},D_{ij}^{s},H_{ij}^{s}\right]=\sum_{n=1}^{3}\int_{z_{n-1}}^{z_{n}}Q_{ij}^{\left(n\right)}\left[1,z,z^{2},{\left(f^{\prime }\left(z\right)\right)}^{2},f\left(z\right),zf\left(z\right),f^{2}\left(z\right)\right]dz$$

Meanwhile, the *δV* produced by external loads for this composite sandwich can be calculated as:

(58) $$\delta V=-\int_{S}F\delta w_{0}dS-\int_{S}\left(N_{xx}^{\mathrm{HT}}\frac{\partial^{2}w_{0}}{\partial x^{2}}+N_{yy}^{\mathrm{HT}}\frac{\partial^{2}w_{0}}{\partial y^{2}}\right)\delta w_{0}dS$$

where, hygrothermal forces $N_{xx}^{HT}$ and $N_{yy}^{HT}$, can be determined according to [48]:

(59) $$\left\{\begin{array}{c} N_{xx}^{\mathrm{HT}}\\ N_{yy}^{\mathrm{HT}} \end{array}\right\}=\sum_{n=1}^{3}\left\{\begin{array}{c} \left(Q_{11}^{\left(n\right)}\alpha_{11}^{\left(n\right)}+Q_{12}^{\left(n\right)}\alpha_{22}^{\left(n\right)}\right)\left(T-T_{0}\right)+\left(Q_{11}^{\left(n\right)}\beta_{11}^{\left(n\right)}+Q_{12}^{\left(n\right)}\beta_{22}^{\left(n\right)}\right)\left(H-H_{0}\right)\\ \left(Q_{21}^{\left(n\right)}\alpha_{11}^{\left(n\right)}+Q_{22}^{\left(n\right)}\alpha_{22}^{\left(n\right)}\right)\left(T-T_{0}\right)+\left(Q_{21}^{\left(n\right)}\beta_{11}^{\left(n\right)}+Q_{22}^{\left(n\right)}\beta_{22}^{\left(n\right)}\right)\left(H-H_{0}\right) \end{array}\right\}dz$$

Substituting Equations (47), (52), and (58) into Equation (46), and then applying the integration by parts, the dynamic equilibrium equations are expressed as:

(60) $$J_{x0}{\ddot{u}}_{0}-J_{x1}\frac{\partial {\ddot{w}}_{0}}{\partial x}+J_{x3}{\ddot{\phi }}_{x}=\frac{\partial N_{xx}}{\partial x}+\frac{\partial N_{xy}}{\partial y}+\frac{1}{R_{x}}\left(\frac{\partial M_{xx}}{\partial x}+\frac{\partial M_{xy}}{\partial y}\right)$$

(61) $$J_{y0}{\ddot{v}}_{0}-J_{y1}\frac{\partial {\ddot{w}}_{0}}{\partial y}+J_{y3}{\ddot{\phi }}_{y}=\frac{\partial N_{yy}}{\partial y}+\frac{\partial N_{xy}}{\partial x}+\frac{1}{R_{y}}\left(\frac{\partial M_{yy}}{\partial y}+\frac{\partial M_{xy}}{\partial x}\right)$$

(62) $$\begin{array}{l} I_{0}{\ddot{w}}_{0}+J_{x1}\frac{\partial {\ddot{u}}_{0}}{\partial x}+J_{y1}\frac{\partial {\ddot{v}}_{0}}{\partial y}-I_{2}\left(\frac{\partial^{2}{\ddot{w}}_{0}}{\partial x^{2}}+\frac{\partial^{2}{\ddot{w}}_{0}}{\partial y^{2}}\right)+I_{4}\left(\frac{\partial {\ddot{\phi }}_{x}}{\partial x}+\frac{\partial {\ddot{\phi }}_{y}}{\partial y}\right)\\ =-\left(\frac{N_{xx}}{R_{x}}+\frac{N_{yy}}{R_{y}}\right)+\frac{\partial^{2}M_{xx}}{\partial x^{2}}+2\frac{\partial^{2}M_{xy}}{\partial x\partial y}+\frac{\partial^{2}M_{yy}}{\partial y^{2}}+F+N_{xx}^{\mathrm{HT}}\frac{\partial^{2}w_{0}}{\partial x^{2}}+N_{yy}^{\mathrm{HT}}\frac{\partial^{2}w_{0}}{\partial y^{2}} \end{array}$$

(63) $$J_{x3}{\ddot{u}}_{0}-I_{4}\frac{\partial {\ddot{w}}_{0}}{\partial x}+I_{5}{\ddot{\phi }}_{x}=\frac{\partial P_{xx}}{\partial x}+\frac{\partial P_{xy}}{\partial y}-S_{xz}$$

(64) $$J_{y3}{\ddot{v}}_{0}-I_{4}\frac{\partial {\ddot{w}}_{0}}{\partial y}+I_{5}{\ddot{\phi }}_{y}=\frac{\partial P_{yy}}{\partial y}+\frac{\partial P_{xy}}{\partial x}-S_{yz}$$

For the impactor, the equation of motion can be expressed as:

(65) $$m_{0}\frac{d^{2}\bar{w}\left(t\right)}{dt^{2}}=F_{c}$$

in which the impactor’s mass is defined by *m*_0_.

## 3. Solution Process

In order to perform a numerical analysis for the dynamic equations at an arbitrary boundary condition, the Galerkin method was introduced, and the Newmark direct integration was applied. The displacements of the functional gradient sandwich shell can be discretized as a sum of a series of functions of the following form according to reference [49]:

(66) $$u_{0}=\sum_{m=1}^{\infty }\sum_{n=1}^{\infty }U_{mn}\left(t\right)\frac{\partial X_{m}\left(x\right)}{\partial x}Y_{n}\left(y\right)$$

(67) $$v_{0}=\sum_{m=1}^{\infty }\sum_{n=1}^{\infty }V_{mn}\left(t\right)X_{m}\left(x\right)\frac{\partial Y_{n}\left(y\right)}{\partial y}$$

(68) $$w_{0}=\sum_{m=1}^{\infty }\sum_{n=1}^{\infty }W_{mn}\left(t\right)X_{m}\left(x\right)Y_{n}\left(y\right)$$

(69) $$\phi_{x}=\sum_{m=1}^{\infty }\sum_{n=1}^{\infty }\Phi_{mn}\left(t\right)\frac{\partial X_{m}\left(x\right)}{\partial x}Y_{n}\left(y\right)$$

(70) $$\phi_{y}=\sum_{m=1}^{\infty }\sum_{n=1}^{\infty }\Psi_{mn}\left(t\right)X_{m}\left(x\right)\frac{\partial Y_{n}\left(y\right)}{\partial y}$$

where *U_mn_*(*t*), *V_mn_*(*t*), *W_mn_*(*t*), *Φ_mn_*(*t*), and *Ψ_mn_*(*t*) are amplitudes that are required to be defined, and *X_m_*(*x*) and *Y_n_*(*y*) are applied to satisfy the equation for an arbitrary boundary condition at defined edges. These spatial basis functions are provided in Table 2. Moreover, the following initial boundary conditions are achieved:

(71) $$\begin{array}{l} U_{mn}\left(0\right)=V_{mn}\left(0\right)=W_{mn}\left(0\right)=\Phi_{mn}\left(0\right)=\Psi_{mn}\left(0\right)=0\\ {\dot{U}}_{mn}\left(0\right)={\dot{V}}_{mn}\left(0\right)={\dot{W}}_{mn}\left(0\right)={\dot{\Phi }}_{mn}\left(0\right)={\dot{\Psi }}_{mn}\left(0\right)=0 \end{array}$$

The initial displacement and velocity of the impactor can be expressed as:

(72) $$\bar{w}\left(0\right)=0,\;\dot{\bar{w}}\left(0\right)=-V_{0}$$

The composite sandwich shell is assumed to have simply supported (*S*) or clamped (*C*) edges, or combinations of them, and they are given as:

Simply supported (*S*):

(73) $$\begin{array}{l} u_{0}=v_{0}=w_{0}=M_{xx}=P_{xx}=0\;\;\mathrm{at}\;x=0,L_{x}\\ u_{0}=v_{0}=w_{0}=M_{yy}=P_{yy}=0\;\;\mathrm{at}\;y=0,L_{y} \end{array}$$

Clamped (*C*):

(74) $$u_{0}=v_{0}=w_{0}=\phi_{x}=\phi_{y}=0\;\;\mathrm{at}\;x=0,L_{x};y=0,L_{y}$$

Substituting Equations (66)–(70) into Equations (60)–(64), the motion of the composite shell can be expressed as:

(75) $$\left[M\right]\left\{\ddot{X}\right\}+\left[C\right]\left\{\dot{X}\right\}+\left[K\right]\left\{X\right\}=\left\{F\right\}$$

where [*M*], [*C*], [*K*], {*X*}, and {*F*} are the matrices of mass, damping, and stiffness, and the vectors of deformation and equivalent external load, respectively. In the presented analysis, the symmetric laminate sandwich shell is constructed; thus, these matrices are all symmetric. The mass matrices and stiffness matrices are provided in Appendix A.

The Rayleigh’s proportional damping model is employed in this analysis, incorporating a damping matrix that consists of both mass- and stiffness-proportional terms. This damping matrix can be expressed as follows:

(76) $$\left[C\right]=\alpha_{D}\left[M\right]+\beta_{D}\left[K\right]$$

where *α_D_* and *β_D_* are Rayleigh’s damping parameters, and those two parameters can be determined according to the viscoelastic material’s damping ratio *ξ* and the two fundamental frequencies *ω_i_* and *ω_j_* of the composite laminated shell, as shown in the following equation as:
where the damping coefficients *α_D_* and *β_D_* are associated with the Rayleigh’s proportional damping model and can be determined based on the damping ratio *ξ* and the two natural frequencies of the structure *ω_i_* and *ω_j_*. The relationship between these parameters can be expressed as:
  (77) $$\alpha_{D}=\frac{2\xi \omega_{i}\omega_{j}}{\omega_{i}+\omega_{j}},\;\beta_{D}=\frac{2\xi }{\omega_{i}+\omega_{j}}$$

In the presented investigation, the Young’s modulus of the viscoelastic polymer material is assumed to be complex, as:

(78) $$E^{*\mathrm{VEM}}=E\left(1+i\eta^{\mathrm{VEM}}\right),\;i=\sqrt{-1}$$

where i is the imaginary unit, *η*^VEM^ is the viscoelastic polymer material’s loss factor, and *E* expresses the storage modulus. Then, equations of the free vibration can be derived as an eigenvalue problem, which can be expressed as:

(79) $$\left|\left[K\right]-{\left(\omega^{*}\right)}^{2}\left[M\right]\right|=0$$

where ω* expresses the complex frequency. Furthermore, the natural frequency and damping ratio of the viscoelastic composite sandwich can be achieved as:

(80) ω=Reω∗2, ξ=Imω∗2/Reω∗2

For solving the impact response of the composite laminate shell structure, the Newmark direct integration algorithm is utilized, which discretizes differential Equation (75) into a time domain. Check Ref. [42] for more detail about the preformation of the Newmark direct integration algorithm. 

## 4. Results and Discussion

### 4.1. Validation

Case 1: the validation of dimensionless natural frequencies of simply supported Al/Al_2_O_3_ functionally graded plates and shells. The current analysis model can be applied to research the dynamic characteristics of a plate structure if the radiuses *R_x_* = *R_y_* = ∞. In this case, the geometric parameter of the structure is *L_x_* = *L_y_* = 10*h* and the material properties of the structure are shown in Table 3. As shown in Table 4, the dimensionless frequencies of the structures obtained by the present method are almost the same as those obtained in Ref. [51]. Hence, the effectiveness of the present model can be validated.

Case 2: the validation of dimensionless natural frequencies of simply supported Al/Al_2_O_3_ functionally graded plates and shells with a negative Poisson’s ratio honeycomb core. In this case, the face-sheets of the composite sandwich structure are made of Al/Al_2_O_3_ functionally graded material and the honeycomb core layer is made of Al. The material properties of the structure are listed in Table 1. The geometric parameters of the structure are *L_x_* = *L_y_* = 20*h*, *h*_c_/*h*_f_ = 2, *η*_1_ = 2, *η*_3_ = 0.0138571, *θ* = −55°. Table 5 provides the dimensionless natural frequencies of the structures for different power law indexes. We can find that the natural frequencies obtained by the current model achieve reasonable consistency with those obtained in Ref. [52] based on FSDT. The present results are about 3% less than those in the literature. The main reason for this difference is that the stiffness of the structure predicted by FSDT is larger than that by predicted by HSDT in the present work, and thus the frequencies calculated based on FSDT will be larger.

**Figure 5:**
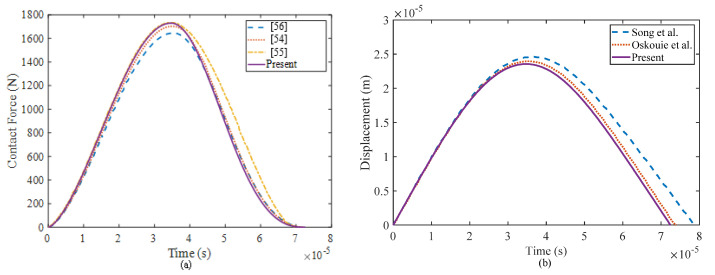
Comparison of the low-velocity impact responses of a simply supported isotropic square plate: (**a**) contact force and (**b**) displacement of the impactor [54,55,56].

Case 3: In previous literature, there is a lack of available results regarding the impact responses of a composite sandwich shell with a multilayer negative Poisson’s ratio honeycomb core. Therefore, in order to verify the accuracy and effectiveness of the current method, a degenerated model is presented. The example focuses on the low-velocity impact problem of a simply supported square isotropic plate and is discussed in detail in the references cited [53,54]. The isotropic plate in consideration has dimensional parameters of *a × b × h* = 200 × 200 × 8 mm. It experiences an impact load from a spherical impactor with a radius of 10 mm and an initial velocity of 1 m/s. Both the plate and impactor share the same material properties, including a mass density of *ρ* = 7810 kg/m^−3^, Young’s modulus of *E* = 206.8 GPa, and Poisson’s ratio of *ν* = 0.3. Figure 5 presents a comparison of the time history of the contact force and displacement of the impactor throughout the impact duration.

The comparison of the results shows that the contact force obtained through the present method is close to the results in Ref. [55] during the loading stage and in Refs. [54,56]. during the unloading stage. In the early stage, there is minimal discrepancy in the displacements of the impactor between these results. However, as the unloading stage progresses, deviations gradually increase due to the accumulation of the differences in the contact force over time. Overall, these comparisons indicate that the solutions obtained by the present method are in excellent agreement with those reported in the previous literature. The slight differences observed can be attributed to the use of different theories, such as the first-order shear deformation plate theory in Ref. [56] and the classical plate theory in Ref. [54], as well as variations in numerical solution procedures.

**Figure 6:**
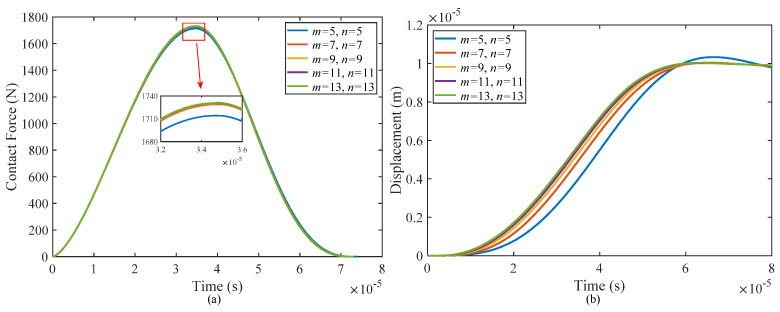
Galerkin method convergence investigation: (**a**) contact force and (**b**) displacement at the impact point.

In the Galerkin method, the number of terms of the spatial function is finite and must be truncated. With an increase in the number of selected spatial function terms, the calculation results gradually converge to the exact solution, but meanwhile, the amount of calculation also increases. Therefore, on the premise of ensuring the convergence of the results, the appropriate number of truncated items should be selected to achieve a balance between computational accuracy and computational efficiency. Figure 6 presents the convergence analysis of the Galerkin method used in the present study. As the truncation numbers (*m* and *n*) increase, the difference in calculation results decreases. It can be observed that convergence is achieved when the truncation numbers reach *m* = 11 and *n* = 11. Therefore, for the remainder of the paper, we selected these two truncated coefficients to ensure accurate and reliable results.

### 4.2. Parametric Analysis

In this subsection, a detailed investigation is performed on several parameters that influence the response of the sandwich shell during impact. These parameters include the temperature, moisture, power law index, thickness of the honeycomb wall, and initial velocity of the impactor. Each parameter is studied thoroughly to understand its influence on the behavior of the sandwich shell under impact conditions. The effects of varying these parameters are analyzed and discussed in depth to provide a comprehensive understanding of their role in determining the impact responses of the sandwich shell. Unless otherwise stated, the geometric parameter of the sandwich shell is *L_x_* = *L_y_ =* 0.8 m and the core-to-face layer thickness ratio is *h*_c_/*h*_f_ = 10. The thickness ratio of the multilayer honeycomb is 1:8:1 and the geometric parameters of the honeycomb core are *η*_1_ = 3, *η*_3_ = 0.1, *θ* = −30°. The radius and initial velocity of the impactor are *R*_0_ = 15 mm and *V*_0_ = 5 m/s. The parameters of the Si_3_N_4_/SUS304 functionally graded material are listed in Table 1. Its material properties are determined through references [14,57,58,59]:

The material of the face layers and honeycomb are the same:

$$E=206\left(1.02-0.0928e^{0.00357\Delta T}\right)\;\mathrm{GPa},\;\nu =0.29,\;\rho =7800\;\mathrm{kg}/m^{3},\;\alpha =10.8\times 10^{-6}\;K^{-1}$$

The material properties of VEM are as follows:

$$E^{\mathrm{VEM}}=\left(3.51-0.03T-0.142H\right)\;\mathrm{GPa},\;\nu^{\mathrm{VEM}}=0.34,\;\rho^{\mathrm{VEM}}=1200\;\mathrm{kg}/m^{3},\;\eta^{\mathrm{VEM}}=0.125$$

$$E^{*\mathrm{VEM}}=E^{\mathrm{VEM}}\left(1+i\eta^{\mathrm{VEM}}\right),\;i=\sqrt{-1}$$

$$\alpha^{\mathrm{VEM}}=45\left(1+0.001\Delta T\right)\times 10^{-6}\;K^{-1},\;\beta^{\mathrm{VEM}}=2.68\times 10^{-3}\;{\mathrm{wt}\%}^{-1}$$

The material properties of the impactor are:

$$E^{\mathrm{imp}}=210\;\mathrm{GPa},\;\nu^{\mathrm{imp}}=0.3,\;\rho^{\mathrm{imp}}=7880\;\mathrm{kg}/m^{3}$$

#### 4.2.1. Effect of Temperature

**Figure 7:**
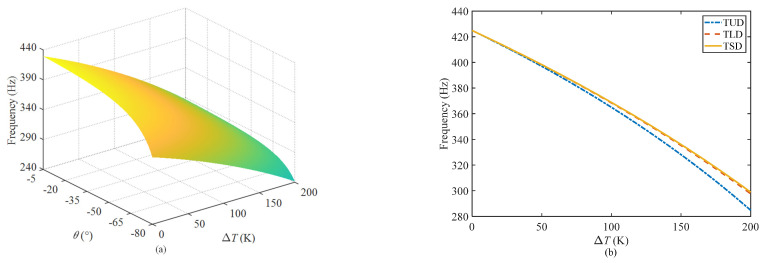
The natural frequencies of the composite sandwich shell: (**a**) variation with the inclined angle and temperature and (**b**) variation with temperature for *θ* = −30° under three different distribution patterns.

Figure 7a shows the changes in the natural frequencies of the composite sandwich shell with the temperature and the inner angle of the honeycomb under the uniform distribution pattern of temperature. The natural frequency of the structure decreases with an increase of temperature and the inclined angle. The main reason is that the elastic modulus of the material will decrease with an increase of temperature. Within the range of inclined angle illustrated in Figure 7a, the equivalent moduli of the honeycomb structure decreased with the increase of the inclined angle, resulting in a decrease of the overall stiffness of the structure, and the natural frequency decreased accordingly.

Figure 7b shows the differences in the natural frequencies with the temperature under three temperature distribution patterns when the inner angle of the honeycomb is *θ* = −30°. This figure indicates that the natural frequency of the structure experiences the most significant decrease under a uniform distribution of temperature and the least under a sinusoidal distribution of temperature. Combined with the expressions of the different temperature distribution patterns, the temperature change at each spatial position along the thickness direction of the structure is Δ*T* in the case of a uniform distribution. However, for the other two distribution patterns, the temperature changes along the thickness direction from 0 to Δ*T* in a certain gradient, and the total temperature change is small, resulting in less influence on the natural frequency of the structure.

**Figure 8:**
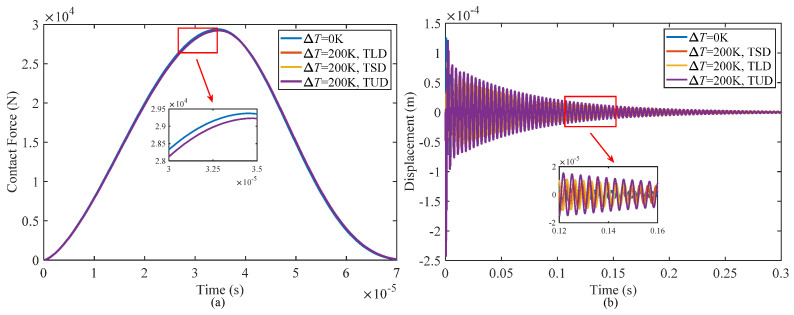
Influence of different temperature distribution patterns on the impact responses of the composite structure: (**a**) contact force, and (**b**) displacements at the impact point.

Figure 8 illustrates the effect of temperature variations on the contact force and displacement of sandwich shells at the contact point, considering three different temperature distribution patterns for Δ*T* = 200 K and Δ*T* = 0 K. The peak value of the contact force will decrease with a temperature rise, but there are no significant differences among the contact force curves under the three temperature distribution patterns. In view of the characteristics of the temperature distribution in this study, the temperature at the top surface of the structure remains constant under the three distribution patterns, and the mechanical properties of both the outermost surface of the sandwich shell and the impactor are also the same, resulting in an unchanging contact stiffness during the low-velocity impact. Hence, the impact process shows minimal variation in both the contact force and contact duration time across the different temperature distribution patterns.

Although there is no significant difference in the contact force, the displacement of the sandwich shell at the impact point exhibits noticeable variations. As the temperature increases, the displacement at the impact point increases dramatically and also shows distinct differences under different temperature distribution patterns. The maximum displacement amplitude occurs when the temperature is uniformly distributed, followed by a linear distribution and a minimum when the temperature shows a sinusoidal distribution. In order to further describe the attenuation characteristics and decay time of the structure during impact, the peak point of the displacement time curve for the wave at the indentation point can be fitted by an exponential function in terms of [60]:

(81) $$y=Ae^{-Bt}$$

Then, the decay time is denoted as *t*_1_, representing the time required for the displacement amplitude to decrease to 1/*e* (approximately 36.8%) of its maximum value [60]. This value can be determined by utilizing a fitted function that accurately describes the decay behavior of the displacement amplitude over time. By analyzing the fitted function, the specific time *t*_1_ can be obtained, providing valuable insight into the rate of decay and the overall behavior of the system.

As shown from the data in Table 6, the displacement response at the impact point of the structure is nearly identical under both linear temperature distribution and sinusoidal distribution conditions, with only a 3.65% difference in displacement amplitude and a 2.41% difference in decay time. However, it is noteworthy that a uniform temperature distribution leads to a more severe deterioration of damping performance of the structure. The displacement amplitude increases by 22.84% compared with that under the condition of a linear distribution, and the decay time is prolonged by 19.83%.

#### 4.2.2. Effect of Moisture

**Figure 9:**
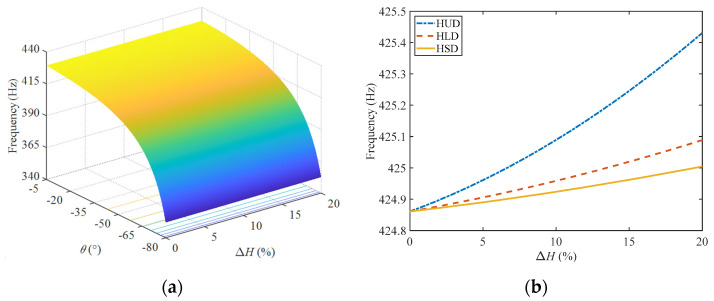
The natural frequencies of the composite sandwich shell: (**a**) variation with the inclined angle and moisture and (**b**) variation with moisture for *θ* = −30° under three different distribution patterns.

When moisture is uniformly distributed along the thickness direction, the natural frequency of the composite sandwich shell varies with moisture and the inclined angle of the honeycomb, as shown in Figure 9a. With an increase of the inclined angle, the natural frequency of the sandwich shell gradually decreases, especially in the range of −55° to −80°, where an increase in the inclined angle will lead to a more significant reduction in the natural frequency. 

Figure 9b illustrates the changes trend of the natural frequency of a structure with moisture in three moisture distribution patterns when the inner angle *θ* = −30°. With an increase of moisture, the natural frequency of this composite structure slightly increases. On the one hand, the mechanical parameters of the viscoelastic polymer materials in the structure are sensitive to moisture changes; on the other hand, only the middle layer of the negative Poisson’s ratio honeycomb is made of viscoelastic polymer material, and its composition proportion in the structure is not high, and thus it has little influence on the performance of the structure. With an increase of moisture, the elastic modulus of the viscoelastic polymer materials will decrease slightly, and the stiffness of the structure should have decreased. However, due to the negative Poisson’s ratio effect of the honeycomb core, the tensile stiffness and bending stiffness of the structure will change, and finally the natural frequency will increase slightly.

**Figure 10:**
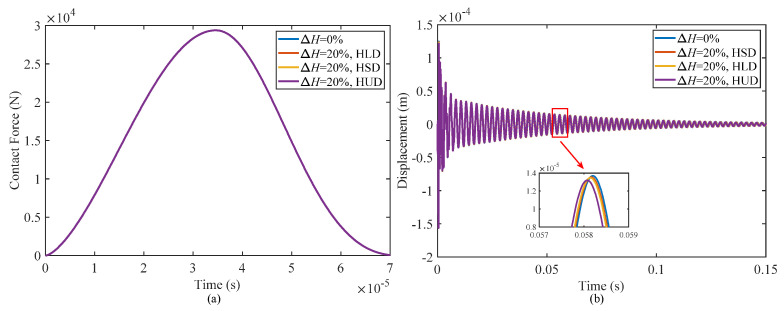
Effect of different moisture distribution patterns on the impact responses of the structure: (**a**) contact force and (**b**) displacement of the structure at the impact point.

Figure 10 illustrates the impact responses of a sandwich shell under different moisture distribution patterns, taking into account moisture variations of ΔH = 20% and ΔH = 0%. The figure depicts that the influence of moisture on the contact force and displacement at the impact point is minimal. However, there is a slight reduction in displacement at the impact point as the moisture content increases. This study examined three moisture distribution patterns, namely a uniform distribution, linear distribution, and sinusoidal distribution. The results indicate that the uniform distribution pattern leads to the greatest decrease in displacement, followed by the linear distribution pattern, while the sinusoidal distribution pattern causes the least decrease. This behavior is consistent with the influence of temperature, suggesting a similar relationship between these two parameters.

The results of function fitting on the displacement time curves of the structure at the impact point under different moisture distributions are listed in Table 7. Compared with the case of reference moisture, when Δ*H* = 20% and the moisture is uniformly distributed, the displacement amplitude decreases the most, reaching 9.23%, and the decay time extends 35.62% correspondingly. It can be seen that although the moisture change has no significant effect on the stiffness of the structure, it will significantly affect the damping performance of the structure.

#### 4.2.3. Effect of Power Law Index

**Figure 11:**
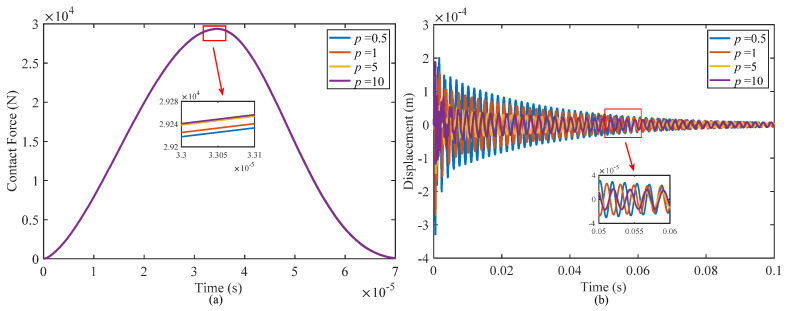
Effect of different power law indexes on the dynamics responses of the structure: (**a**) contact force and (**b**) displacement at the impact point.

Figure 11 illustrates how the power index of the functionally graded face-sheets affects the contact force and displacement of a sandwich shell at the impact point. While the contact stiffness remains constant for different power law indices during the impact, variations in the structural stiffness and damping performance lead to different levels of indentation on the structure’s surface, resulting in distinct peak values of the contact force. With an increase in the power law index, there is a slight elevation in the peak contact force, but the contact duration remains unaltered throughout the impact process.

With an increase of the power law index, the volume fraction of ceramic material at the same position on the face-sheets will decrease, resulting in a reduction in both the stiffness and mass of the face-sheets. Consequently, the natural frequency of the structure will also decrease. As the stiffness of the core layer is lower than that of the face-sheets, the deformation primarily occurs within the core layer. Therefore, the displacement of the structure at the impact point will decrease when the stiffness of the face-sheets decreases.

Table 8 shows the results of function fitting of the displacement time curve for different power law indexes. As evidenced by data in the table, with an increase of the power law index, the displacement amplitude of the impact point decreases, and the fitting index *B* also decreases, resulting in prolongation of the vibration attenuation. It can be inferred that increasing the power law index will mitigate the deformation of the structure while slowing down the vibration attenuation. Hence, the selection of an appropriate power law index in structural design is crucial, as it not only prevents excessive deformation of the structure but also enhances its damping performance. This facilitates rapid dissipation of the vibration energy and minimizes the generation of sound radiation noise caused by vibration.

#### 4.2.4. Effect of the Thickness of Honeycomb Wall

**Figure 12:**
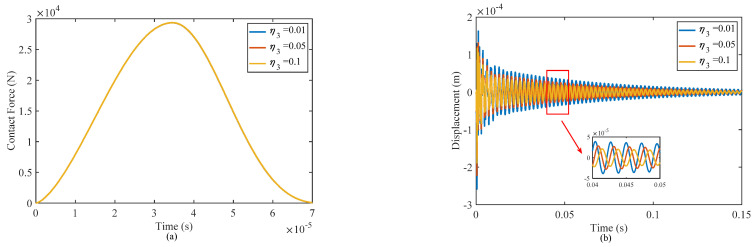
Effect of different thicknesses of the honeycomb wall on responses of the structure: (**a**) contact force and (**b**) displacement at the impact point.

Figure 12 present the influence of the honeycomb wall thickness of the core layer on the contact force and displacement at the impact point under a low-velocity impact. When the thickness of the honeycomb walls increases, the equivalent elastic moduli of the core layer will increase, resulting in an increase in stiffness of the sandwich structure, and then the resistance to deformation of the structure will be enhanced. Additionally, there is a slight increase in the peak contact force and a decrease in the displacement amplitude at the impact point under a low-velocity impact.

With the increase of honeycomb wall thickness, the volume fraction of the viscoelastic polymer material increases, the damping performance of sandwich shell structure is enhanced, and then the vibration attenuation time is also shortened, which is consistent with the fitting data in Table 9. Therefore, the stiffness and damping performance of the structure can be improved by increasing the thickness of the honeycomb wall, and the vibration of the structure can be effectively suppressed.

#### 4.2.5. Effect of Initial Velocity of the Impactor

**Figure 13:**
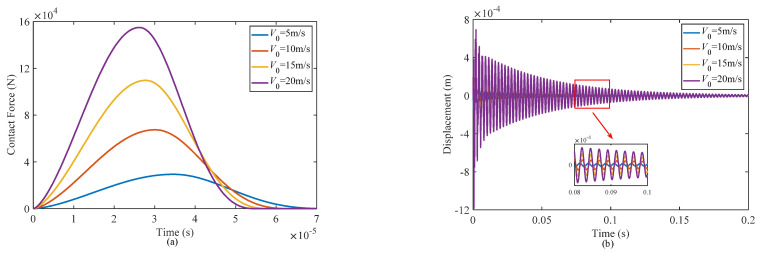
Effect of different initial velocities of the impactor on the responses of the structure: (**a**) contact force and (**b**) displacement at the impact point.

Figure 13 depicts the time history curves of the contact force and displacement of the sandwich shell at the impact point for different initial velocities of the impactor. With an increase of the initial velocity of the impactor, the impact energy acting on the structure increases, leading to a significant increase in the contact force and a decrease in the contact duration time. When the initial velocity of the impactor is doubled, the peak value of the contact force increases by approximately 2.3 times.

An increase in the initial velocity of the impactor results in a significant rise in the displacement amplitude of the structure at the impact point. Table 10 provides function fitting results for different initial velocities of the impactor, showing that while the structure remains unchanged, there is a variation in the displacement amplitude. Interestingly, the fitting parameter B remains nearly identical across different velocities, indicating a consistent relationship between the impactor’s initial velocity and the displacement amplitude. Furthermore, the vibration attenuation time remains constant, suggesting that it is independent of the initial velocity of the impactor.

## 5. Conclusions

This study focused on the design of a new functionally graded composite sandwich doubly curved shell with a multilayer negative Poisson’s ratio honeycomb core. The dynamic responses of this structure when subjected to low-velocity impacts in hygrothermal environments were investigated. The dynamic equilibrium equations for the structure under different temperature and moisture distribution patterns were derived using higher-order shear deformation theory and Hamilton’s principle. To numerically solve the dynamic problem, the Galerkin method and the Newmark direct integration scheme were employed to discretize the differential dynamic equations in the space and time domains, respectively. Subsequently, a parametric study was conducted to analyze the effects of various factors on the contact force and the displacement at the impact point of the structure. Based on this analysis, the following five conclusions can be obtained:

(1) Increasing temperature will reduce the contact force during the impact process, and then the damping performance of the structure will deteriorate, resulting in an increase of displacement at the impact point and prolongation of the vibration decay time. There was no significant difference between the contact force under three different temperature distribution patterns, but the damping performance of the structure decreased most seriously when the temperature was uniformly distributed throughout the thickness.
(2) Increasing moisture has almost no influence on the contact force during the impact process, but it will slightly reduce the displacement of the structure at the impact point and increase the vibration attenuation time. The structural stiffness is slightly improved due to the negative Poisson’s ratio effect, but the damping performance is significantly decreased.
(3) The power law index has a direct effect on the mass and stiffness of the face-sheets of the structure. As the power law index increases, the contact force during the impact process increases slightly and the deformation resistance of the structure is enhanced, leading to a reduction of displacement at the impact point; however, the damping performance of the structure also decreases, and then the vibration dissipation time becomes longer.
(4) Increasing the thickness of the honeycomb wall will increase the impact contact force, improving the overall stiffness and energy dissipation characteristics of the structure. Therefore, increasing the thickness of the honeycomb wall is an effective approach to improve both the stiffness and damping performance of the structure.
(5) The contact force and central deflection of the structure experience a significant increase when the initial velocity of the impactor is raised. This amplification is attributed to the higher impact energy associated with the increased velocity.

Furthermore, it is important to note that in the current study, the analysis focused on small and elastic deformations of the composite laminated shell structure. This implies that once the contact force is released, the deformation of the laminate shell will promptly recover. Therefore, the calculation methodologies utilized are not applicable for determining large and plastic deformations. Additionally, two thermal distribution configurations are considered, namely the uniform distribution and sinusoidal distribution. However, in practical engineering scenarios, the thermal field distribution tends to be highly complex and intricate.

## Figures and Tables

**Table 1:** Temperature-dependent coefficients for Si_3_N_4_ and SUS304 [42].

Material	Property	*P*_0_	*P*_1_	*P*_2_	*P*_3_
Ceramic (Si_3_N_4_)	*E* (Pa)	3.4843 × 10^11^	−3.0700 × 10^−4^	2.1600 × 10^−7^	−8.9460 × 10^−11^
	*ρ* (kg/m^3^)	2370	0	0	0
	ν	0.24	0	0	0
	*α* (K^−1^)	5.8723 × 10^−6^	9.0950× 10^−4^	0	0
Metal (SUS304)	*E* (Pa)	2.0104 × 10^11^	3.0790 × 10^−4^	−6.5340 × 10^−7^	0
	*ρ* (kg/m^3^)	8166	0	0	0
	ν	0.3262	−2.0020 × 10^−4^	3.7970 × 10^−7^	0
	*α* (K^−1^)	1.2330 × 10^−5^	8.0860 × 10^−4^	0	0

**Table 2:** Boundary conditions determination [50].

Functions	Boundary Conditions		
	SSSS	CSSS	CSCS
*X_m_*(*x*)	sin(*λx*)	sin(*λx*)(cos(*λx*)-1)	sin(*λx*)(cos(*λx*)-1)
*Y_n_*(*y*)	sin(*μy*)	sin(*μy*)	sin(*μy*)(cos(*μy*)-1)

Note: λ = mπx/a, μ = nπy/b.

**Table 3:** Material properties of functionally graded structures.

Material	*E* (GPa)	*ρ* (kg/m^3^)	ν
Al	69	2707	0.3
Al_2_O_3_	380	3800	0.3

**Table 4:** Comparison of dimensionless natural frequencies of Al/Al_2_O_3_ functionally graded structures.

Structure	Method	*p* = 0	*p* = 0.5	*p* = 1.0	*p* = 4.0	*p* = 10.0
Plate (*L_x_*/*R_x_* = *L_y_*/*R_y_* = 0)	Ref. [51]	0.0577	0.0490	0.0442	0.0382	0.0366
	Present	0.0577	0.0490	0.0442	0.0381	0.0364
Cylindrical shell (*L_x_*/*R_x_* = 0.5, *L_y_*/*R_y_* = 0)	Ref. [51]	0.0617	0.0527	0.0477	0.0407	0.0385
	Present	0.0617	0.0527	0.0477	0.0405	0.0383
Spherical shell (*L_x_*/*R_x_* = *L_y_*/*R_y_* = 0.5)	Ref. [51]	0.0746	0.0646	0.0588	0.0491	0.0455
	Present	0.0746	0.0646	0.0588	0.0490	0.0453

**Table 5:** Comparison of dimensionless natural frequencies of Al/Al_2_O_3_ functionally graded structures with a negative Poisson’s ratio honeycomb core.

Structure	Method	*p* = 0.5	*p* = 1.0	*p* = 5.0	*p* = 10.0
Plate (*L_x_*/*R_x_* = *L_y_*/*R_y_* = 0)	Ref. [52]	0.0181	0.0172	0.0137	0.0123
	Present	0.0177	0.0167	0.0133	0.0119
Cylindrical shell (*L_x_*/*R_x_* = 0.5, *L_y_*/*R_y_* = 0)	Ref. [52]	0.0186	0.0176	0.0140	0.0125
	Present	0.0182	0.0171	0.0136	0.0123
Spherical shell (*L_x_*/*R_x_* = *L_y_*/*R_y_* = 0.5)	Ref. [52]	0.0201	0.0189	0.0150	0.0135
	Present	0.0197	0.0185	0.0146	0.0132

**Table 6:** Exponential function parameters and decay times are determined for various temperature distribution patterns.

Distribution Pattern	*A* (m)	*B*	*t*_1_ (s)
Δ*T* = 0 K	4.874 × 10^−5^	21.77	0.0306
Δ*T* = 200 K, TSD	6.594 × 10^−5^	15.08	0.0566
Δ*T* = 200 K, TLD	6.844 × 10^−5^	14.93	0.0580
Δ*T* = 200 K, TUD	8.407 × 10^−5^	14.01	0.0695

**Table 7:** Exponential function parameters and decay times obtained through curve fitting.

Distribution Pattern	*A* (m)	*B*	*t*_1_ (s)
Δ*H* = 0%	4.874 × 10^−5^	21.77	0.0306
Δ*H* = 20%, HSD	4.708 × 10^−5^	20.52	0.0310
Δ*H* = 20%, HLD	4.707 × 10^−5^	20.62	0.0309
Δ*H* = 20%, HUD	4.424 × 10^−5^	20.49	0.0415

**Table 8:** Fitted exponential function parameters and decay times for different power law indexes.

*p*	*A* (m)	*B*	*t*_1_ (s)
0.5	1.631 × 10^−4^	32.91	0.0273
1	1.154 × 10^−4^	28.65	0.0292
5	4.874 × 10^−5^	21.85	0.0298
10	5.393 × 10^−5^	20.59	0.0305

**Table 9:** Fitted exponential function parameters and decay times for different thicknesses of the honeycomb wall.

*η*_3_	*A* (m)	*B*	*t*_1_ (s)
0.01	8.053 × 10^−5^	16.42	0.0377
0.5	6.480 × 10^−5^	18.84	0.0323
0.1	5.393 × 10^−5^	20.59	0.0305

**Table 10:** Fitted exponential function parameters and decay times for different initial velocities of the impactor.

*V*_0_ (m/s)	*A* (m)	*B*	*t*_1_ (s)
5	5.393 × 10^−5^	20.59	0.0305
10	1.610 × 10^−4^	20.25	0.0319
15	3.393 × 10^−4^	20.09	0.0339
20	5.405 × 10^−4^	20.03	0.0351

## Data Availability

Data are contained within the article.

## Appendix A

The components of the mass and stiffness matrices in Equation (75) can be given in the following forms:

(A1) $$\left[M\right]=\left[\begin{array}{ccccc} M_{11} & M_{12} & M_{13} & M_{14} & M_{15}\\ M_{21} & M_{22} & M_{23} & M_{24} & M_{25}\\ M_{31} & M_{32} & M_{33} & M_{34} & M_{35}\\ M_{41} & M_{42} & M_{43} & M_{44} & M_{45}\\ M_{51} & M_{52} & M_{53} & M_{54} & M_{55} \end{array}\right],\;\left[K\right]=\left[\begin{array}{ccccc} K_{11} & K_{12} & K_{13} & K_{14} & K_{15}\\ K_{21} & K_{22} & K_{23} & K_{24} & K_{25}\\ K_{31} & K_{32} & K_{33} & K_{34} & K_{35}\\ K_{41} & K_{42} & K_{43} & K_{44} & K_{45}\\ K_{51} & K_{52} & K_{53} & K_{54} & K_{55} \end{array}\right]$$

The components of the mass matrix in Equation (75) can be given in the following forms:

(A2) $$\begin{array}{l} M_{11}=J_{x0}\chi_{10}^{\left(1\right)},\;M_{12}=0,\;M_{13}=-J_{x1}\chi_{10}^{\left(1\right)},\;M_{14}=J_{x3}\chi_{10}^{\left(1\right)},\;M_{15}=0\\ M_{21}=0,\;M_{22}=J_{y0}\chi_{01}^{\left(2\right)},\;M_{23}=-J_{y1}\chi_{01}^{\left(2\right)},\;M_{24}=0,\;M_{25}=J_{y3}\chi_{01}^{\left(2\right)}\\ M_{31}=J_{x1}\chi_{20}^{\left(3\right)},\;M_{32}=J_{y1}\chi_{02}^{\left(3\right)},\;M_{33}=I_{0}\chi_{00}^{\left(3\right)}-I_{2}\left(\chi_{20}^{\left(3\right)}+\chi_{02}^{\left(3\right)}\right)\\ M_{34}=I_{4}\chi_{20}^{\left(3\right)},\;M_{35}=I_{4}\chi_{02}^{\left(3\right)}\\ M_{41}=J_{x3}\chi_{10}^{\left(1\right)},\;M_{42}=0,\;M_{43}=-I_{4}\chi_{10}^{\left(1\right)},\;M_{44}=I_{5}\chi_{10}^{\left(1\right)},\;M_{45}=0\\ M_{51}=0,\;M_{52}=J_{y3}\chi_{01}^{\left(2\right)},\;M_{53}=-I_{4}\chi_{01}^{\left(2\right)},\;M_{54}=0,\;M_{55}=I_{5}\chi_{01}^{\left(2\right)} \end{array}$$

The components of the stiffness matrix in Equation (75) can be obtained as follows:

(A3) $$\begin{array}{l} K_{11}=-\left(A_{11}+2r_{x}B_{11}+r_{x}^{2}D_{11}\right)\chi_{30}^{\left(1\right)}-\left(A_{66}+2r_{x}B_{66}+r_{x}^{2}D_{66}\right)\chi_{12}^{\left(1\right)}\\ K_{12}=-\left(A_{12}+A_{66}+r_{x}r_{y}\left(D_{12}+D_{66}\right)+\left(r_{x}+r_{y}\right)\left(B_{12}+B_{66}\right)\right)\chi_{12}^{\left(1\right)}\\ K_{13}=\left(B_{11}+r_{x}D_{11}\right)\chi_{30}^{\left(1\right)}+\left(B_{12}+2B_{66}+r_{x}\left(D_{12}+2D_{66}\right)\right)\chi_{12}^{\left(1\right)}\\ \;\;-\left(r_{x}A_{11}+r_{y}A_{12}+r_{x}r_{y}B_{12}+r_{x}^{2}B_{11}\right)\chi_{10}^{\left(1\right)}\\ K_{14}=-\left(B_{11}^{s}+r_{x}D_{11}^{s}\right)\chi_{30}^{\left(1\right)}-\left(B_{66}^{s}+r_{x}D_{66}^{s}\right)\chi_{12}^{\left(1\right)}\\ K_{15}=-\left(B_{12}^{s}+B_{66}^{s}+r_{x}\left(D_{12}^{s}+D_{66}^{s}\right)\right)\chi_{12}^{\left(1\right)}\\ K_{21}=-\left(A_{21}+A_{66}+\left(r_{x}+r_{y}\right)\left(B_{21}+B_{66}\right)+r_{x}r_{y}\left(D_{21}+D_{66}\right)\right)\chi_{21}^{\left(2\right)}\\ K_{22}=-\left(A_{22}+2r_{y}B_{22}+r_{y}^{2}D_{22}\right)\chi_{03}^{\left(2\right)}-\left(A_{66}+2r_{y}B_{66}+r_{y}^{2}D_{66}\right)\chi_{21}^{\left(2\right)}\\ K_{23}=\left(B_{22}+r_{y}D_{22}\right)\chi_{03}^{\left(2\right)}+\left(B_{21}+2B_{66}+r_{y}\left(D_{21}+2D_{66}\right)\right)\chi_{21}^{\left(2\right)}\\ \;\;-\left(r_{x}A_{21}+r_{y}A_{22}+r_{x}r_{y}B_{21}+r_{y}^{2}B_{22}\right)\chi_{01}^{\left(2\right)}\\ K_{24}=-\left(B_{21}^{s}+B_{66}^{s}+r_{y}\left(D_{21}^{s}+D_{66}^{s}\right)\right)\chi_{21}^{\left(2\right)}\\ K_{25}=-\left(B_{66}^{s}+r_{y}D_{66}^{s}\right)\chi_{21}^{\left(2\right)}-\left(B_{22}^{s}+r_{y}D_{22}^{s}\right)\chi_{03}^{\left(2\right)}\\ K_{31}=\left(r_{x}A_{11}+r_{y}A_{21}+r_{x}r_{y}B_{21}+r_{x}^{2}B_{11}\right)\chi_{20}^{\left(3\right)}\\ \;\;-\left(B_{11}+r_{x}D_{11}\right)\chi_{40}^{\left(3\right)}-\left(B_{21}+2B_{66}+r_{x}\left(D_{21}+2D_{66}\right)\right)\chi_{22}^{\left(3\right)}\\ K_{32}=\left(r_{x}A_{12}+r_{y}A_{22}+r_{x}r_{y}B_{12}+r_{y}^{2}B_{22}\right)\chi_{02}^{\left(3\right)}\\ \;\;-\left(B_{22}+r_{y}D_{22}\right)\chi_{04}^{\left(3\right)}-\left(B_{12}+2B_{66}+r_{y}\left(D_{12}+2D_{66}\right)\right)\chi_{22}^{\left(3\right)}\\ K_{33}=D_{11}\chi_{40}^{\left(3\right)}+D_{22}\chi_{04}^{\left(3\right)}+\left(D_{12}+D_{21}+4D_{66}\right)\chi_{22}^{\left(3\right)}-\left(r_{x}B_{11}+r_{y}\left(B_{12}+B_{21}\right)-N_{xx}^{\mathrm{HT}}\right)\chi_{20}^{\left(3\right)}\\ \;\;-\left(r_{x}\left(B_{12}+B_{21}\right)+2r_{y}B_{22}-N_{yy}^{\mathrm{HT}}\right)\chi_{02}^{\left(3\right)}+\left(r_{x}^{2}A_{11}+r_{x}r_{y}\left(A_{12}+A_{21}\right)+r_{y}^{2}A_{22}\right)\chi_{00}^{\left(3\right)}\\ K_{34}=-D_{11}^{s}\chi_{40}^{\left(3\right)}-\left(D_{21}^{s}+2D_{66}^{s}\right)\chi_{22}^{\left(3\right)}+\left(r_{x}B_{11}^{s}+r_{y}B_{21}^{s}\right)\chi_{20}^{\left(3\right)}\\ K_{35}=-\left(D_{12}^{s}+2D_{66}^{s}\right)\chi_{22}^{\left(3\right)}-D_{22}^{s}\chi_{04}^{\left(3\right)}+\left(r_{x}B_{12}^{s}+r_{y}B_{22}^{s}\right)\chi_{02}^{\left(3\right)} \end{array}$$

(A4) $$\begin{array}{l} K_{41}=-\left(B_{11}^{s}+r_{x}D_{11}^{s}\right)\chi_{30}^{\left(1\right)}-\left(B_{66}^{s}+r_{x}D_{66}^{s}\right)\chi_{12}^{\left(1\right)}\\ K_{42}=-\left(B_{12}^{s}+B_{66}^{s}+r_{y}\left(D_{12}^{s}+D_{66}^{s}\right)\right)\chi_{02}^{\left(1\right)}\\ K_{43}=D_{11}^{s}\chi_{30}^{\left(1\right)}+\left(D_{12}^{s}+2D_{66}^{s}\right)\chi_{12}^{\left(1\right)}-\left(r_{x}B_{11}^{s}+r_{y}B_{12}^{s}\right)\chi_{10}^{\left(1\right)}\\ K_{44}=-H_{11}^{s}\chi_{30}^{\left(1\right)}-H_{66}^{s}\chi_{12}^{\left(1\right)}+A_{55}^{s}\chi_{10}^{\left(1\right)},\\ K_{45}=-\left(H_{12}^{s}+H_{66}^{s}\right)\chi_{12}^{\left(1\right)}\\ K_{51}=-\left(B_{21}^{s}+B_{66}^{s}+r_{x}\left(D_{21}^{s}+D_{66}^{s}\right)\right)\chi_{21}^{\left(2\right)}\\ K_{52}=-\left(B_{66}^{s}+r_{y}D_{66}^{s}\right)\chi_{21}^{\left(1\right)}-\left(B_{22}^{s}+r_{y}D_{22}^{s}\right)\chi_{03}^{\left(1\right)}\\ K_{53}=\left(D_{21}^{s}+2D_{66}^{s}\right)\chi_{21}^{\left(1\right)}+D_{22}^{s}\chi_{03}^{\left(1\right)}-\left(r_{x}B_{21}^{s}+r_{y}B_{22}^{s}\right)\chi_{01}^{\left(1\right)}\\ K_{54}=-\left(H_{21}^{s}+H_{66}^{s}\right)\chi_{21}^{\left(1\right)}\\ K_{55}=-H_{22}^{s}\chi_{03}^{\left(1\right)}+A_{44}^{s}\chi_{01}^{\left(1\right)}-H_{66}^{s}\chi_{21}^{\left(1\right)} \end{array}$$

where *r_x_* and *r_y_* are two constants, *r_x_* = 1/*R_x_*, *r_y_* = 1/*R_y_*. *χ* is the integration constant, which is related to the geometry dimensions and boundary conditions of the structure in terms of:

(A5) $$\left[\chi_{pq}^{\left(1\right)},\chi_{pq}^{\left(2\right)},\chi_{pq}^{\left(3\right)}\right]=\int_{0}^{a}\int_{0}^{b}\frac{\partial^{p}X_{m}}{\partial x}\frac{\partial^{q}Y_{n}}{\partial y}\left[\frac{\partial X_{m}}{\partial x}Y_{n},X_{m}\frac{\partial Y_{n}}{\partial y},X_{m}Y_{n}\right]dxdy\left(p,q=0,1,2,3,4\right)$$
